# No additional risk of congenital anomalies after first-trimester dydrogesterone use: a systematic review and meta-analysis

**DOI:** 10.1093/hropen/hoae004

**Published:** 2024-01-23

**Authors:** Alexander Katalinic, Maria R Noftz, Juan A Garcia-Velasco, Lee P Shulman, John N van den Anker, Jerome F Strauss III

**Affiliations:** Institute for Social Medicine and Epidemiology, University of Luebeck, Luebeck, Germany; Institute for Social Medicine and Epidemiology, University of Luebeck, Luebeck, Germany; IVI RMA Global Research Alliance, Madrid, Spain; Department of Obstetrics and Gynaecology, Rey Juan Carlos University, Madrid, Spain; Division of Clinical Genetics, Department of Obstetrics & Gynecology, Feinberg School of Medicine of Northwestern University, Chicago, IL, USA; Division of Clinical Pharmacology, Children’s National Hospital, Washington, DC, USA; Pediatric Pharmacology and Pharmacometrics Research Center, University Children’s Hospital Basel, Basel, Switzerland; Department of Obstetrics and Gynecology, Perelman School of Medicine, University of Pennsylvania, Philadelphia, PA, USA

**Keywords:** progesterone, meta-analysis, congenital abnormality, recurrent miscarriage, miscarriage, ART

## Abstract

**STUDY QUESTION:**

Is exposure to dydrogesterone a risk factor for congenital anomalies when given in the first trimester for recurrent/threatened pregnancy loss or as luteal support in assisted reproductive technology (ART)?

**SUMMARY ANSWER:**

Dydrogesterone, when given in the first trimester for recurrent/threatened pregnancy loss or as luteal support in ART, is not a relevant additional risk factor for congenital anomalies.

**WHAT IS KNOWN ALREADY:**

Despite large clinical trials and meta-analyses that show no association between dydrogesterone and congenital anomalies, some recently retracted publications have postulated an association with teratogenicity. Dydrogesterone is also often rated as less safe than bioidentical progestins.

**STUDY DESIGN, SIZE, DURATION:**

A systematic review was conducted according to a pre-specified protocol with searches on Medline, Embase, Cochrane Central Register of Controlled Trials (CENTRAL), and Clinicaltrials.gov. The search was limited to human studies, with no restrictions on language, geographical region, or date. The search algorithm used a PICO (Population, Intervention, Comparison, Outcome)-style approach combining both simple search terms and medical subject heading terms. As congenital anomalies are mostly reported as secondary outcomes, the search term ‘safety’ was added.

**PARTICIPANTS/MATERIALS, SETTING, METHODS:**

Interventional study and observational study (OS) designs were eligible for inclusion. Inclusion criteria were: women >17 years old treated for threatened miscarriage, recurrent pregnancy loss, and/or ART; the use of dydrogesterone in the first trimester compared with placebo, no treatment or other interventions; and reporting of congenital anomalies in newborns or infants ≤12 months old (primary outcome). Two authors (A.K., M.R.N.) independently extracted the following data: general study information, study population details, intervention and comparator(s), and frequencies of congenital anomalies (classification, time of determination, and type). Risk of bias focused on the reporting of congenital malformations and was assessed using the Cochrane Risk of Bias Tool Version 2 or the ROBINS-I tool. The GRADEproGDT platform was used to generate the GRADE summary of findings table.

**MAIN RESULTS AND THE ROLE OF CHANCE:**

Of the 897 records retrieved during the literature search, 47 were assessed for eligibility. Nine studies were included in the final analysis: six randomized controlled trials (RCTs) and three OSs. Among the RCTs, three had a low risk and three a high risk of bias. Two of the OSs were considered to have a serious risk of bias and one with critical risk of bias and was excluded for the evidence syntheses. The eight remaining studies included a total of 5070 participants and 2680 live births from 16 countries. In the meta-analysis of RCTs only, the overall risk ratio (RR) was 0.92 [95% CI 0.55; 1.55] with low certainty. When the two OSs were included, the overall RR was 1.11 [95% CI 0.73; 1.68] with low certainty.

**LIMITATIONS, REASONS FOR CAUTION:**

The studies included in the analysis do not report congenital anomalies as the primary outcome; reporting of congenital anomalies was often not standardized.

**WIDER IMPLICATIONS OF THE FINDINGS:**

This systematic literature review and meta-analysis provide clear reassurance to both clinicians and patients that dydrogesterone is not associated with congenital anomalies above the rate that might be expected due to environmental and genetic factors. The results of this work represent the highest current level of evidence for the question of congenital anomalies, which removes the existing uncertainty caused by poor quality and retracted studies.

**STUDY FUNDING/COMPETING INTEREST(S):**

Editorial support was provided by Highfield Communication Consultancy, Oxford, UK, sponsored by Abbott Products Operations AG, Allschwil, Switzerland. A.K., J.A.G.-V., L.P.S., J.N.v.d.A., and J.F.S. received honoraria from Abbott for preparation and participation in an advisory board. J.A.G.-V. received grants and lecture fees from Merck, Organon, Ferring, Gedeon Richter, and Theramex. M.R.N. has no conflicts of interest. J.N.v.d.A. and J.A.G.-V. have no other conflicts of interest. A.K. received payment from Abbott for a talk at the IVF Worldwide congress on 22 September 2023. J.F.S. has received grants from the National Institutes of Health, royalties/licences from Elsevier and Prescient Medicine (SOLVD Health), consulting fees from Burroughs Wellcome Fund (BWF) and Bayer, honoraria from Magee Women's Research Institute, Wisconsin National Primate Research Centre, University of Kansas and Oakridge National Research Laboratory, Agile, Daiichi Sankyo/American Regent, and Bayer, and travel support to attend meetings for the International Academy of Human Reproduction (IAHR). J.F.S. has patents related to diagnosis and treatment of PCOS and prediction of preterm birth. J.F.S. participates on advisory boards for SOLVD Health, Wisconsin National Primate Research Centre, and FHI360, was the past President board member of the Society for Reproductive Investigation, has a leadership role for the following organizations: Scientific Advisory Board, SOLVD Health, EAB Chair for contraceptive technology initiative, FHI360, EAB member, Wisconsin National Primate Research Centre, Advisory Board for MWRI Summit, Chair of BWF NextGen Pregnancy Research Panel, Medical Executive Committee at the Howard, and Georgeanna Jones Foundation, and is Vice President, IAHR. L.P.S. has received consulting fees from Shield Pharmaceuticals, Scynexis, Organon, Natera, Celula China, AiVF, Agile, Daiichi Sankyo, American Regent, and Medicem, honoraria from Agile, Daiichi Sankyo/American Regent, and Bayer, and travel support from BD Diagnostics. L.P.S. participates on the data safety monitoring board for Astellas and is a Chair of DSMB for fezolinetant. Abbott played no role in the funding of the study or in study design, data collection, data analysis, data interpretation, or writing of the report.

**TRIAL REGISTRATION NUMBER:**

PROSPERO 2022 CRD42022356977.

WHAT DOES THIS MEAN FOR PATIENTS? This study looks at all the available scientific evidence on taking dydrogesterone in the first trimester of pregnancy and the risk of birth deformities.

Progesterone is a hormone that prepares the uterus to accept and maintain an embryo. Women undergoing IVF are given a progesterone supplement for a short time after the embryo has been transferred. Women who have had recurrent miscarriage or who are at risk of miscarriage may also be treated with progesterone in their first trimester. All progesterone products come from plant-based sources and are identical to the natural progesterone produced by the human body. Dydrogesterone originates from the plant but has been exposed to ultraviolet light to increase delivery of the bioactive hormone when taken by mouth.

It is important to ensure that any medicine taken during pregnancy does not harm the mother or the baby. Pregnant women should be aware that, unfortunately, birth deformities do occur for many reasons. This study provides the best possible, clear reassurance that taking dydrogesterone adds no relevant additional risk for birth deformities above the rate that might be expected for all progestogen drugs or due to environmental and genetic factors.

## Introduction

Dydrogesterone was specifically developed to avoid androgenic effects of synthetic progestins and to provide high bioavailability when taken orally (Schindler, 2009; Rižner *et al.*, 2011; Griesinger *et al.*, 2019) and is a treatment option for women with recurrent pregnancy loss (RPL) or at risk of miscarriage in the first trimester. As with all progesterone products used for this indication, dydrogesterone is manufactured from a plant source, the wild yam (Schindler *et al.*, 2008; Schindler, 2009; Mount Sinai Health Library, 2023). Dydrogesterone has been in clinical use for over 40 years.

Although large clinical trials and meta-analyses have not provided evidence for an association between dydrogesterone taken in the first trimester for these indications and congenital anomalies, some publications have reported an association with teratogenicity, most of which have since been retracted due to methodological flaws (Retraction Watch Database, n.d.). Additionally, playing on the default belief that natural entities are better, dydrogesterone is often rated apparently less healthy and less safe than natural entities (Li and Cao, 2020). One of the first examples of the bias for the preferential use of ‘bioidentical” hormones was published in 1998 when Baron *et al.* found that obstetricians selected a natural over a synthetic hormone replacement therapy for a hypothetical patient, even when the two therapies were described as identical (Baron *et al.*, 1998; Li and Cao, 2020; Ji *et al.*, 2023).

Although the most recent ESHRE Guidance on the management of RPL provides a conditional recommendation that there is insufficient evidence to recommend the use of progesterone to improve live birth rates in women with repeated pregnancy loss and luteal phase insufficiency (ESHRE, 2022), there is a biologically plausible rationale for providing progestogenic support during the luteal phase (Babalioğlu *et al.*, 1996; Li *et al.*, 2000; Carp, 2018). Nevertheless, it is common in clinical practice to provide luteal phase support for the prevention of miscarriage and in ART (Griesinger *et al.*, 2019; Ovarian Stimulation TEGGO *et al.*, 2020; Devall *et al.*, 2021).

The aim of this study was to supplement and update our previously published scoping review (Katalinic *et al.*, 2022) by providing a complete and systematic review of the available literature to determine whether exposure to dydrogesterone in the first trimester is a risk factor for congenital anomalies.

## Methods

We conducted this systematic review according to a pre-specified protocol that was registered in the international prospective registry for systematic reviews (PROSPERO), published prior to data analysis (CRD 42022356977), and reported in accordance with the PRISMA (Preferred Reporting Items for Systematic Reviews and Meta-Analyses) 2020 checklist (Page *et al.*, 2021).

### Eligibility criteria

Publications on interventional (randomized controlled trials [RCTs]) and observational (cohort and case–control studies) study designs were eligible for inclusion. Patients had to be women older than 17 years and treated for threatened miscarriage, RPL, and/or ART; possible interventions were the use of dydrogesterone in the first trimester; and comparators were placebo, no treatment, or interventions other than dydrogesterone. The main outcome was major congenital anomalies in newborns or infants (up to 12 months of age), as reported by the authors. If possible, congenital anomalies were rated using suitable coding systems such as the European network of population-based registries for the epidemiological surveillance of congenital anomalies (EUROCAT, 2023; https://eu-rd-platform.jrc.ec.europa.eu/eurocat_en). Studies not reporting any congenital anomalies in newborns or infants were excluded.

### Search strategy

Searches were conducted on Medline, Embase, and Cochrane Central Register of Controlled Trials (CENTRAL) from inception to 28 September 2022 to identify studies for inclusion and on Clinicaltrials.gov to identify any ongoing trials or unpublished studies. The search was limited to human studies, but no restrictions on language, geographical region, or date were applied. The search algorithm used a PICO-style approach combining both simple search terms and medical subject heading terms, with the help of Boolean Operators OR, AND, and NOT (Supplementary Table S1). As congenital anomalies are mostly reported as additional (secondary) outcomes rather than as a primary outcome, the term ‘safety’ was added as a search term. A full list of search terms can be found in the study protocol published on PROSPERO. Reference lists of relevant articles were searched for additional publications.

### Study selection

Two reviewers (A.K., M.R.N.) independently screened all retrieved references for inclusion based on title and abstract. The same reviewers independently assessed the full texts of potentially eligible studies. Non-English abstracts and articles were translated by an automatic translation software (www.deepL.com). The Covidence^®^ software was used throughout this whole process to organize and classify the articles. We resolved disagreement during the review by discussion or, if needed, with the help of a third reviewer.

### Data collection

The following information was extracted from each eligible study: general study information (e.g. authors, publication year, study design, aim, funding, conflicts); study population details (sample size, demographic data, body mass index, gravida, parity, number of pregnancies); details of the study intervention and comparison (type, dose, application, duration); and outcomes of interest (frequencies of congenital anomalies, classification, time of determination and type). Two reviewers (A.K., M.R.N.) independently extracted data from each eligible publication using an adapted standardized data collection form in the Covidence^®^ software.

### Risk of bias assessment

Based on the extracted data, the quality of the eligible studies was assessed using the Cochrane Risk of Bias Tool Version 2 (Sterne *et al.*, 2019) for RCTs and the ROBINS-I tool (Sterne *et al.*, 2016) for observational studies (OSs). Clarifications regarding details of the methods of included studies were sought from published protocols, statistical analysis plans, or trial reports, when available. Risk of bias was adjudicated as low only if all domains were assessed as low risk of bias. Congenital malformations were not the primary outcome in most studies; however, this outcome was the focus for the bias assessment of the studies to ensure that the quality estimation of the evidence was not compromised. Two reviewers (A.K., M.R.N.) independently assessed the risk of bias for each of the included studies. Disagreements were resolved by discussion and, if needed, by involving one of the co-authors.

### Data synthesis

The main outcome variable was the presence of a congenital malformation in exposed and non-exposed live births. All identified RCTs and OSs with non-critical bias studies were included. The risk ratio (RR) was used to measure effect. For meta-analysis, we used a random-effect model with Mantel–Haenszel estimator, because the populations and the indications for dydrogesterone administration must be considered heterogeneous. CIs (95% CI) were presented, and statistical heterogeneity was assessed using Tau^2^, *I*^2^, and Chi^2^ statistics. Heterogeneity was substantial if *I*^2^ was >30%, Tau^2^ >0, or *P*-value for Chi^2^ statistics was <0.1. Even if no substantial heterogeneity was observed, we carried out two subgroup analyses/sensitivity analyses: (i) overall study quality: high versus low quality and (ii) study design: RCT versus cohort studies.

When dealing with missing data, where possible, review authors extracted data on all participants randomized, with preference for data from intention-to-treat analyses rather than per protocol data. It was initially planned to examine publication bias using funnel plots, but the pre-defined threshold of ≥10 studies was not reached (Sterne *et al.*, 2011). Data synthesis and visualization were performed using R (R version 4.1.3; library: meta; procedure: metabin [metaprop for prevalence]).

### Grading the quality of evidence

The Grading of Recommendation Assessment, Development and Evaluation (GRADE) approach was used to assess the overall quality of evidence for the main outcome measure (The GRADE Working Group *et al.*, 2013), and the GRADEproGDT platform (https://www.gradepro.org/) generated the GRADE summary of findings table.

### Prevalence of congenital anomalies for dydrogesterone-exposed live births stratified by the different types of studies

In addition to the main aim of the study, a meta-analysis was performed on the prevalence of congenital anomalies for dydrogesterone-exposed live births stratified by the different types of studies. The prevalence of malformations was operationalized by the number of malformations among live-born children, analogous to the identified controlled trials. For this analysis, we also included three studies that were initially excluded from our search strategy due to failing the eligibility criteria (single-arm cohort studies or controlled cohort studies in which dydrogesterone was given in all treatment arms). The study characteristics and assessments for these studies are recorded separately (Supplementary Table S2).

## Results

**Figure 1.**
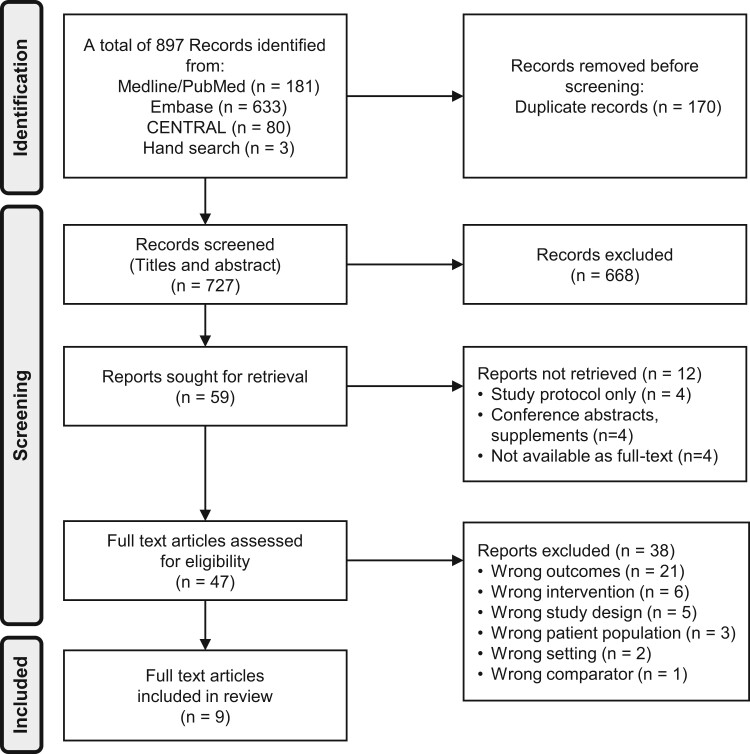
**PRISMA (Preferred reporting Items for Systematic Reviews and Meta-Analyses) flow diagram of results of search and reasons for exclusion of studies**.

A total of 897 records were retrieved during the literature search, of which 47 were assessed for eligibility (Fig. 1). The studies that were subsequently excluded are listed in Supplementary File S1. Only nine studies fulfilled the inclusion criteria: six randomized trials (El-Zibdeh, 2005; El-Zibdeh and Yousef, 2009; Pandian, 2009; Tournaye *et al.*, 2017; Griesinger *et al.*, 2018; Chan *et al.*, 2021) and three OSs (Zaqout *et al.*, 2015; Vuong *et al.*, 2021; Xu *et al.*, 2021). The characteristics of these studies are shown in Tables 1 and 2.

**Table 1.** Characteristics of randomized controlled studies that fulfilled the inclusion criteria.

Study and year	Study design	Country	Indication	Age of women (years)^a^	No randomized (intervention/control), ITT	Intervention (dose and duration)	Control (dose and duration)	No live birth (intervention/control), ITT	Congenital anomalies	
									Coding/classification	When determined
Chan *et al.* (2021)	RCT, DB	Hong Kong SAR, China	Threatened miscarriage first trimester	*Intervention*: 31.3 (±4.3) *Control*: 30.8 (±4.3)	203/203	Dydrogesterone 40 mg, followed by 30 mg/d until 12th week of gestation or bleeding stopped	Placebo (oral)	165/169	No information	Not mentioned
El-Zibdeh (2005)	Quasi-RCT, OL	Jordan	Unexplained recurrent spontaneous abortion	*Overall*: 20–24 (22%) 25–29 (39%) 30–34 (37%)	82/98	Dydrogesterone 20 mg/d up to 12th week of gestation	hCG 5000 IU every 4 days or no treatment until 12th week of gestation	71/75	No information	Not mentioned
El-Zibdeh and Yousef (2009)	Quasi-RCT, OL	Jordan	Threatened miscarriage first trimester	*Intervention*: 20–24 (25.6%) 25–29 (39.5%) 30–34 (25.6%) ≥35 (9.3%) *Control*: 20–24 (26.7%) 25–29 (36.7%) 30–34 (23.3%) ≥35 (13.3%)	86/60	Dydrogesterone 20 mg/d for 1 week or more after bleeding stopped or earlier if severe bleeding occurred	No treatment	71/45	No information	Not mentioned
Pandian (2009)	RCT, OL	Malaysia	Threatened miscarriage first trimester	*Intervention*: <20 (20%) 21–29 (50%) ≥30 (30%) *Control*: <20 (18%) 21–29 (58%) ≥30 (24%)	96/95	Dydrogesterone 40 mg, followed by 20 mg/d until 16th week of gestation	Bed rest only	84/68	No information	Not mentioned
Griesinger *et al.* (2018)	RCT, OL, NI	Multicentre	Luteal phase support in IVF	*Overall*: 31.7 (±4.5)	494/489	Dydrogesterone (oral), 30 mg/d until 12th week of gestation	MVP gel 8% (vaginal)	205/188	Specified as TEAEs of special interest: congenital, familial, and genetic disorders	At delivery
Tournaye *et al.* (2017)	RCT, TB, NI	Multicentre	Luteal phase support in IVF	*Overall*: 32.5 (±4.4)	497/477	Dydrogesterone (oral) 30 mg/d + Placebo (vaginal) until 12th week of gestation	MVP 600 mg/d + Placebo (oral) until 12th week of gestation	213/158	Specified as TEAEs of special interest: congenital, familial, and genetic disorders	At delivery and at 2-month follow-up

Data are presented as mean age (standard deviation) or age groups (relative frequency).

ITT: intention-to-treat-analyses; CHD, congenital heart defect; DB, double-blind; hCG, human chorionic gonadotrophin; IU, international units; IVF, *in vitro* fertilisation; MVP, micronised vaginal progesterone; NI, non-inferiority; OL, open label; RCT, randomized controlled trial; TB, triple-blind; TEAE, treatment-emergent adverse event.

**Table 2.** Characteristics of observational studies that fulfilled in the inclusion criteria.

Study and year	Study design	Country	Indication	Age of women (years)^a^	No randomized (intervention/control), ITT^a^	Intervention (dose and duration)	Control (dose and duration)	No live birth (intervention/control), ITT	Congenital anomalies	
									Coding/classification	When determined
Vuong *et al.* (2021)	Prospective cohort study	Vietnam	Luteal phase support in FET	*Intervention*: 31.33 (±4.50) *Control*: 31.36 (±4.42)	732/632	Dydrogesterone 20 mg/day + MVP 800 mg/d until 7 weeks of gestation with positive pregnancy test	MVP 800 mg/day until 7 weeks of gestation when pregnancy test positive	339/261	Not specified	Not mentioned
Xu *et al.* (2021)	Retrospective cohort study (1:3 matching)	China	Luteal phase support for HRT–FET cycles	*Intervention*: 32.00 (±4.68) *Control*: 32.05 (±4.92)	208/624 cycles	Dydrogesterone 20 mg/day + progesterone sustained-release gel (vaginal) 90 mg/day for 10–11 weeks	Progesterone IM 40 mg/day for 10–11 weeks	139/429	Not specified	Not mentioned
Zaqout *et al.* (2015) ^a^	Case–control study	Palestine	Impact of dydrogesterone exposure on occurrence of CHD	*Cases*: <20 (6%) 20–35 (90.1%) >35 (4%) *Controls*: <20 (0.5%) 20–35 (94%) >35 (5.5%)	202 (cases)/200 (controls)	Children born with CHD (cases)	Children born without CHD (controls)	Dydrogesterone exposure yes/no: Cases 75/127 Controls 36/164	Mitchells definition of CHD (Mitchell 1971)	Not mentioned

Excluded from analysis due to critical bias.

CHD, congenital heart defect; DB, double-blind; hCG, human chorionic gonadotrophin; HRT-FET, hormone replacement therapy in congenital embryo transfer; IM, intramuscular; ITT, intention-to-treat-analyses; IU, international units; IVF, *in vitro* fertilisation; MVP, micronised vaginal progesterone; NI, non-inferiority; OL, open label; TB, triple-blind; TEAE, treatment-emergent adverse event.

**Figure 2.**
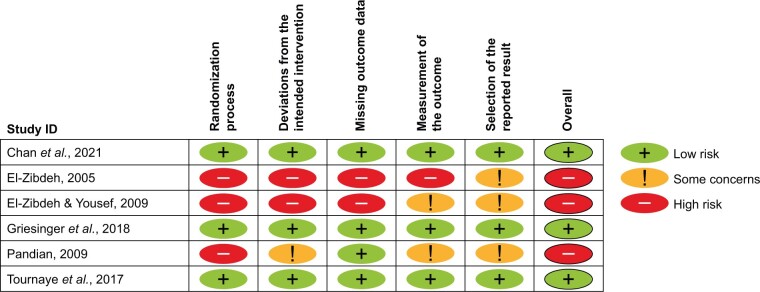
**Risk of bias for the randomized controlled trials (RCTs) using the Cochrane Risk of Bias Tool (Sterne *et al.*, 2019).** See Supplementary Fig. S1 for the risk of bias items presented as percentages across all included RCTs.

All identified studies underwent risk of bias assessment. Among the six RCTs, three were considered to have a low risk of bias (Pandian, 2009; Tournaye *et al.*, 2017; Griesinger *et al.*, 2018) and three to have a high risk of bias (El-Zibdeh, 2005; El-Zibdeh and Yousef, 2009; Chan *et al.*, 2021); for details, see Fig. 2 and Supplementary Fig. S1. Two of the OSs were considered to have a serious risk of bias (Vuong *et al.*, 2021; Xu *et al.*, 2021) and one to have critical risk of bias, as there were serious deficits in the validation of dydrogesterone intake, likely recall and selection bias (Zaqout *et al.*, 2015); see Table 3. Due to the critical risk of bias, this study was not included in the subsequent evidence synthesis.

**Table 3.** Summary of risk of bias in non-randomized studies of interventions (ROBINS-I) tool (Sterne *et al.*, 2016).

Study	Confounding	Selection	Intervention classification	Deviation from intervention	Missing data	Measurement of outcome	Selection of reported results	Overall
Vuong *et al.* (2021)	Serious	Low	Low	Low	Low	Serious	Low	**Serious**
Xu *et al.* (2021)	Serious	Moderate	Low	Low	Low	Serious	Low	**Serious**
Zaqout *et al.* (2015)	Serious	Critical	Serious	Moderate	Moderate	Serious	Serious	**Critical**

The eight remaining studies had a total of 5070 participants and 2680 live births from 16 countries (Australia, Austria, Belgium, China (including Hong Kong), Finland, Germany, India, Israel, Jordan, Malaysia, Russian Federation, Singapore, Spain, Thailand, Ukraine, Vietnam).

**Figure 3.**
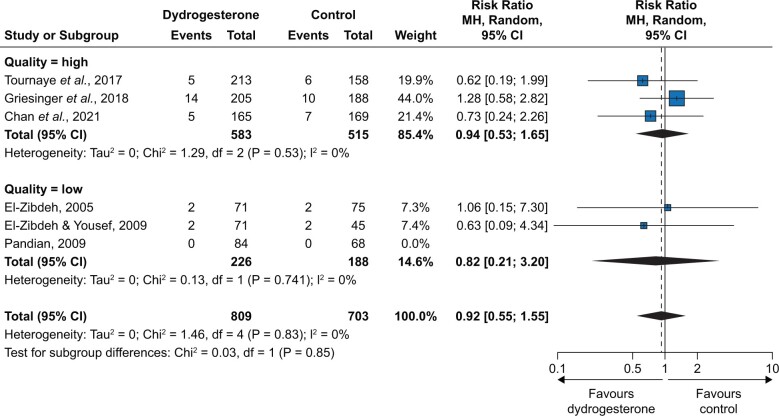
**Results of meta-analysis of randomized controlled trials, stratified by overall study quality (high versus low).** MH, Mantel–Haenszel.

**Figure 4.**
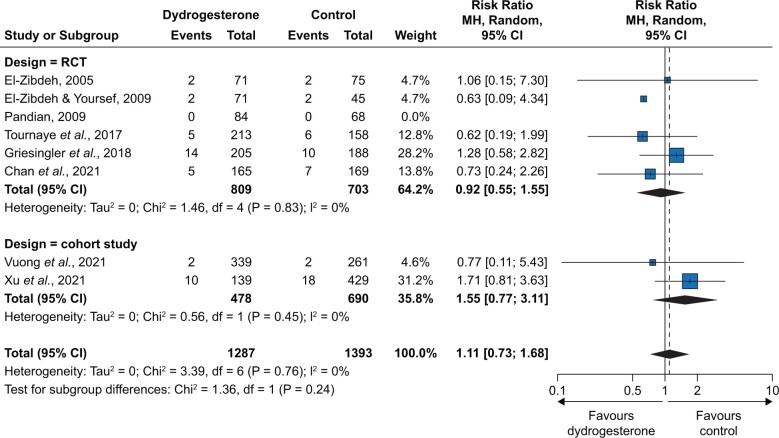
**Results of meta-analysis of randomized controlled trials (RCTs) and cohort studies, stratified by the study design (RCT versus cohort study).** MH, Mantel–Haenszel.

In the meta-analysis of RCTs, the overall RR was 0.92 [95% CI 0.55; 1.55] with a low certainty (Fig. 3), with an RR of 0.94 [0.53; 1.65] for high-quality-rated studies and an RR of 0.82 [0.21; 3.20] for low-quality-rated studies. When the two OSs rated as having non-critical bias were included in the meta-analysis, the overall RR was 1.11 [0.73; 1.68] (low certainty) (Fig. 4). Table 4 summarizes the overall findings for RCTs according to the GRADE approach. The absolute risk for congenital anomalies was 38/1000 live births without and 35/1000 live births with dydrogesterone exposure in the first trimester (non-significant difference). A pooled summary of findings table for RCTs and OSs together leads to a comparable result (Supplementary Table S3).

**Table 4.** Summary of overall findings for RCTs only.

‘With dydrogesterone’ compared to ‘without dydrogesterone’ in the first trimester of pregnancy for the indications of threatened miscarriage or ART						
Patient or population: ART or threatened miscarriage						
Setting: clinical						
Intervention: dydrogesterone in first trimester						
Comparison: no dydrogesterone in first trimester						
Outcomes	Anticipated absolute effects* (95% CI)		Relative effect (95% CI)	No. of participant (studies)	Certainty of the evidence (GRADE)	Comments
	Risk without dydrogesterone	Risk with dydrogesterone				
Congenital anomalies (RCTs only)	38 per 1000	35 per 1000 (21; 60) 3 fewer (17 fewer to 21 more)	RR 0.92 (0.55; 1.55)	1512 (6 studies)	⨁⨁◯◯ Low^a^^,b^	Rate of congenital anomalies is not significantly increased in live births exposed to dydrogesterone in the first trimester

**GRADE Working Group grades of evidence**

High certainty: we are very confident that the true effect lies close to that of the estimate of the effect.

Moderate certainty: we are moderately confident in the effect estimate: the true effect is likely to be close to the estimate of the effect, but there is a possibility that it is substantially different.

Low certainty: our confidence in the effect estimate is limited: the true effect may be substantially different from the estimate of the effect.

Very low certainty: we have very little confidence in the effect estimate: the true effect is likely to be substantially different from the estimate of effect.

The risk in the intervention group (and its 95% CI) is based on the assumed risk in the comparison group and the relative effect of the intervention (and its 95% CI).

Downgraded −1 due to serious limitations in study design (randomization process).

Downgraded −1 due to serious imprecision (wide 95% CIs, low number of events).

RR, risk ratio.

Using all identified information on live births exposed to dydrogesterone in the first trimester of pregnancy (dydrogesterone arm of RCTs or controlled OSs from the meta-analysis above and additional OSs with dydrogesterone without a control group), the overall prevalence of congenital anomalies associated with dydrogesterone in the given indications was 2.5% [95% CI 1.5%; 4.3%] (Supplementary Fig. S2).

## Discussion

This systematic review provides the most complete and robust analysis of the peer-reviewed published literature on the safety of dydrogesterone in live births when given in the first trimester to women with threatened or recurrent miscarriage. It supplements the 2021 Cochrane review of progesterone used in the first trimester in women suffering from RPL or at risk of miscarriage (Devall *et al.*, 2021), which included only one publication on dydrogesterone and confirms the results of a previous scoping review by the same authors (Katalinic *et al.*, 2022). When the data from RCTs only were assessed, the meta-analysis demonstrated a small but not significant benefit of dydrogesterone compared with the controls.

The addition of the two OSs resulted in a non-significant benefit of the controls over dydrogesterone. This is not surprising, as OSs are more prone to bias. For example, while it is probable that the outcome (congenital anomalies) is more likely in the dydrogesterone-exposed group than in the control group (due to diagnostic suspicion bias), many other biases may be present. But it has to be mentioned that neither the RCTs only nor RCTs plus OSs indicated a significant effect of dydrogesterone on congenital anomalies. The combined result leads to absolute risks for congenital anomalies of 35/1000 for dydrogesterone use versus 38/1000 in controls and was based on 1512 live births. The difference is three fewer congenital anomalies per 1000 live births with an uncertainty from 17 fewer to 21 more. The level of evidence for this result is graded as low, but currently, it is the best available evidence. The sensitivity analysis including the two OSs leads to a comparable result (95% CI: 9 fewer to 23 more). These results support the conclusion that the rate of congenital anomalies is not significantly increased (or decreased) in live births exposed to dydrogesterone in the first trimester of the pregnancy. Even if one argues that the power of the meta-analysis is too small to detect an effect of dydrogesterone, it can only be speculated whether it would be harmful or beneficial, but very likely the effect would be small. Therefore, when all eight studies were included, we could only conclude that the rate of congenital anomalies might not be significantly increased in live births exposed to dydrogesterone in the first trimester.

When assessing the impact of dydrogesterone on congenital anomalies, we should put the findings in the context of real-world measures of congenital anomalies. The pooled prevalence of congenital anomalies in our study is 2.5% [95% CI 1.5%; 4.3%] for dydrogesterone-exposed live births. This is within the range published by the EUROCAT Network for congenital anomalies in Europe of 2.15% for ‘all anomalies’ in still and live births in 2021 (data for live births only are not presented; excluding still births might lead to an insignificant lower rate) (Moorthie *et al.*, 2018). But, nevertheless, the data imply that there are no clinically relevant differences and support our conclusion that exposure to dydrogesterone in the first trimester does not lead to an increased number of congenital anomalies.

The strength of this analysis is in the selection of studies; however, the number of RCTs and OSs without critical levels of bias was small and this, therefore, limits the strength of evidence. Furthermore, congenital anomalies are rarely a primary outcome and mostly not documented in a standardized way.

Dydrogesterone is one of only two available oral treatment options for the treatment of women at risk of miscarriage or RPL in the first trimester and those undergoing ART. In such a highly motivated population, patient preferences for the route of progestogen administration are likely to be a secondary consideration (Shoham *et al.*, 2021), but it is still important that patients should be offered a safe choice. For some patients, the requirements for vaginal administration (such as how and when to apply) and potential side effects (such as vaginal irritation, discharge, bleeding, and interference with coitus) may impact their effective use (Licciardi *et al.*, 1999; Czajkowski *et al.*, 2007; Barbosa *et al.*, 2016; Shoham *et al.*, 2021; Parveen *et al.*, 2021; Almohammadi *et al.*, 2022).

Given the increasing utilization of ART worldwide, it is critical that clinicians and patients have access to robust and unbiased information about the congenital safety profile of treatment options. In our experience, unfounded concerns about the congenital safety of dydrogesterone continue to circulate. From time to time, published articles may be retracted due to genuine mistakes. However, a retracted article is formally no longer part of the body of science and must not influence clinical decision-making (van der Vet and Nijveen, 2016).

Even though this systematic review, based on RCTs, gave no hint for an increased risk for congenital anomalies, further evidence should be generated to enhance the body of evidence. Pharmacovigilance data could be used as a ‘sign giver’, but the evidence level of such data is limited due to different reasons (such as incomplete reporting, missing risk factor adjustment, etc.). More high-quality evidence is needed. However, as randomized controlled reproductive medicine studies rarely focus on fetal safety as a primary endpoint, there is no evidence at the highest level available for this topic, and there probably never will be. We suggest that more new randomized controlled studies in the field of threatened miscarriage or ART involving dydrogesterone in the first trimester of pregnancy should include standardized assessment of congenital anomalies as a secondary outcome. Further, it should be discussed whether information on luteal supplementation could be added to national and systematic registries for congenital anomalies to be able to estimate the effects of these therapies and, if necessary, their extent on a population basis.

We believe that our systematic review and meta-analysis provide the best possible reassurance to both clinicians and patients alike that dydrogesterone adds no relevant additional risk for congenital anomalies above the rate that might be expected for all progestogens or environmental and genetic factors.

## Supplementary Material

hoae004_Supplementary_Data

## Data Availability

The data underlying this article are available in the article and in its online supplementary material.
