# Supplementary material for: No additional risk of congenital anomalies after first-trimester dydrogesterone use: a systematic review and meta-analysis
Source: Hum Reprod Open. 2024 Jan 23;2024(1):hoae004. doi: 10.1093/hropen/hoae004 (PMC10859181; doi:10.1093/hropen/hoae004)
Supplement: hoae004_Supplementary_Data [file hoae004_supplementary_data.docx]

**Supplementary File 1. List of the 38 studies excluded due to wrong outcomes, study design, intervention, comparator, patient population and/or setting.**

Afridi N, Masood U, Balooch S, Khan S. Comparison of efficacy of oral progesterone and micronized progesterone pessary in reduction of incidence of spontaneous preterm births. *J Ayub Med Coll Abbottabad* 2019;*31*:248–251.

Alizadeh F, Mahmoudinia M, Mirteimoori M, Pourali L, Niroumand S. Comparison of oral dydrogesterone and 17-α hydroxyprogesterone caprate in the prevention of preterm birth. *BMC Pregnancy Childbirth* 2022;*22*:167. doi: 10.1186/s12884-022-04509-1.

Areeruk W, Phupong V. A randomized, double blinded, placebo controlled trial of oral dydrogesterone supplementation in the management of preterm labor. *Sci Rep* 2016;*6*:20638. doi: 10.1038/srep20638.

Artymuk NV, Noskova IN, Tachkova OA. Effectiveness of preconceptional preparation in women with early pregnancy loss. *Akusherstvo i Ginekologiya* (Russian Federation) 2020;*2020*:83–92. [doi: org/10.18565/aig.2020.1.83-92](doi:%20org/10.18565/aig.2020.1.83-92). Available at: <https://en.aig-journal.ru/archive?release=aig>.

Atzmon Y, Aslih N, Estrada D, Bilgory A, Ellenbogen A, Shalom-Paz E. Comparable outcomes using oral dydrogesterone vs. micronized vaginal progesterone in frozen embryo transfer: a retrospective cohort study. *Reprod Sci* 2021;*28*:1874–1881. doi: 10.1007/s43032-020-00376-3.

Bryce C. Treatment of threatened miscarriage with progestogens. *Am Fam Physician* 2019;*100*:279–280

Cessot M, Salle B, Labrune E, Walter O, Benchaib M, Fraison E. Comparaison de la dydrogestérone avec une progestérone micronisée vaginale dans le transfert d’embryon frais en FIV/ICSI [Comparison of oral dydrogesterone with micronized vaginal progesterone in fresh embryo transfert in IVF/ICSI]. *Gynecol Obstet Fertil Senol* 2022;50:462–469. French. doi: 10.1016/j.gofs.2022.03.002.

Chakravarty BN, Shirazee HH, Dam P, Goswami SK, Chatterjee R, Ghosh S. Oral dydrogesterone versus intravaginal micronised progesterone as luteal phase support in assisted reproductive technology (ART) cycles: results of a randomised study*. J Steroid Biochem Mol Biol* 2005;*97*:416–20. doi: 10.1016/j.jsbmb.2005.08.012.

Coomarasamy A, Devall AJ, Cheed V, Harb H, Middleton LJ, Gallos ID, Williams H, Eapen AK, Roberts T, Ogwulu CC et al. A randomized trial of progesterone in women with bleeding in early pregnancy. *N Engl J Med* 2019;*380*:1815–1824. doi: 10.1056/NEJMoa1813730.

Coomarasamy A, Williams H, Truchanowicz E, Seed PT, Small R, Quenby S, Gupta P, Dawood F, Koot YE, Bender Atik R et al. A randomized trial of progesterone in women with recurrent miscarriages. *N Engl J Med* 2015;*373*:2141–2148. doi: 10.1056/NEJMoa1504927.

Doğan Durdağ G, Bektaş G, Türkyılmaz E, Göktepe H, Sönmezer M, Şükür YE, Özmen B, Atabekoğlu C, Berker B, Aytaç R et al. The efficacy of dydrogesterone use to suppress premature luteinizing hormone surge on cycle outcomes in controlled ovarian stimulation. *J Turk Ger Gynecol Assoc* 2021;*22*:293–299. doi: 10.4274/jtgga.galenos.2020.2020.0110.

Ganesh A, Chakravorty N, Mukherjee R, Goswami S, Chaudhury K, Chakravarty B. Comparison of oral dydrogestrone with progesterone gel and micronized progesterone for luteal support in 1,373 women undergoing in vitro fertilization: a randomized clinical study. *Fertil Steril* 2011;95:1961–1965. doi: 10.1016/j.fertnstert.2011.01.148.

Griesinger G, Blockeel C, Tournaye H. Oral dydrogesterone for luteal phase support in fresh in vitro fertilization cycles: a new standard? *Fertil Steril* 2018;*109*:756–762. doi: 10.1016/j.fertnstert.2018.03.034.

Griesinger G, Tournaye H, Macklon N, Petraglia F, Arck P, Blockeel C, van Amsterdam P, Pexman-Fieth C, Fauser BC. Dydrogesterone: pharmacological profile and mechanism of action as luteal phase support in assisted reproduction. *Reprod Biomed Online* 2019;*38*:249–259. doi: 10.1016/j.rbmo.2018.11.017.

Huang J, Xie Q, Lin J, Lu X, Wang N, Gao H, Cai R, Kuang Y. Neonatal outcomes and congenital malformations in children born after dydrogesterone application in progestin-primed ovarian stimulation protocol for IVF: a retrospective cohort study. *Drug Des Devel Ther* 2019;13:2553–2563. doi: 10.2147/DDDT.S210228.

Jiang S, Chen L, Cai R, Kuang Y. A follow-up study on congenital anomalies in 2208 offspring of three years old born after luteal-phase stimulation. *Reprod Biomed Online* 2022;*45*:589–598. doi: 10.1016/j.rbmo.2022.04.007.

Kale AR, Kale AA, Yelikar K. A comparative, randomized control trial in patients of per vaginal bleeding comparing efficacy of oral dydrogesterone versus vaginal progesterone in successful pregnancy outcome for patients with recurrent pregnancy loss. *J Obstet Gynaecol India* 2021;*71*:591–595. doi: 10.1007/s13224-021-01473-2.

Khosravi D, Taheripanah R, Taheripanah A, Tarighat Monfared V, Hosseini-Zijoud SM. Comparison of oral dydrogesterone with vaginal progesterone for luteal support in IUI cycles: a randomized clinical trial. *Iran J Reprod Med* 2015;13:433–438.

Kumar A, Begum N, Prasad S, Aggarwal S, Sharma S. RETRACTED: Oral dydrogesterone treatment during early pregnancy to prevent recurrent pregnancy loss and its role in modulation of cytokine production: a double-blind, randomized, parallel, placebo-controlled trial. *Fertil Steril* 2014;*102*:1357–1363.e3. doi: 10.1016/j.fertnstert.2014.07.1251. Retraction in: *Fertil Steril* 2023;*119*:518.

Lotfalizadeh M, Khademi Z, Maleki A, Najafi MN. Comparison of the duration of pregnancy in administration of progesterone suppository and Duphaston tablet in pregnant women with preterm labor after stopping delivery process. *IJOGI* 2019;*22*:1–11. doi: [10.22038/IJOGI.2019.14184](https://doi.org/10.22038/ijogi.2019.14184)

Lou C, Wang C, Zhao Q, Jin F. Effect of dydrogesterone and progesterone on threatened miscarriage due to corpus luteum insufficiency. *Am J Transl Res* 2021;*13*:4544–4552.

Nadarajah R, Rajesh H, Wong KY, Faisal F, Yu SL. Live birth rates and safety profile using dydrogesterone for luteal phase support in assisted reproductive techniques. *Singapore Med J* 2017;*58*:294–297. doi: 10.11622/smedj.2016080.

Omar MH, Mashita MK, Lim PS, Jamil MA. Dydrogesterone in threatened abortion: pregnancy outcome. *J Steroid Biochem Mol Biol* 2005;*97*:421–425. doi: 10.1016/j.jsbmb.2005.08.013.

Ozer G, Yuksel B, Yucel Cicek OS, Kahraman S. Oral dydrogesterone vs. micronized vaginal progesterone gel for luteal phase support in frozen-thawed single blastocyst transfer in good prognosis patients. *J Gynecol Obstet Hum Reprod* 2021;*50*:102030. doi: 10.1016/j.jogoh.2020.102030.

Pang YY, Ma CL. Real-world pharmacological treatment patterns of patients with threatened miscarriage in China from 2014 to 2020: A cross-sectional analysis. *J Clin Pharm Ther* 2022;*47*:228–236. doi: 10.1111/jcpt.13536.

Patki A, Pawar VC. Modulating fertility outcome in assisted reproductive technologies by the use of dydrogesterone. *Gynecol Endocrinol* 2007;*23* Suppl 1:68–72. doi: 10.1080/09513590701584857.

Pustotina O. Effectiveness of dydrogesterone, 17-OH progesterone and micronized progesterone in prevention of preterm birth in women with a short cervix. *J Matern Fetal Neonatal Med* 2018;*31*:1830–1838. doi: 10.1080/14767058.2017.1330406.

Queisser-Luft A. Dydrogesterone use during pregnancy: overview of birth defects reported since 1977. *Early Hum Dev* 2009;*85*:375–377. doi: 10.1016/j.earlhumdev.2008.12.016.

Hossein Rashidi B, Tarafdari A, Ghazimirsaeed ST, Shahrokh Tehraninezhad E, Keikha F, Eslami B, Ghazimirsaeed SM, Jafarabadi M. Comparison of dydrogesterone and GnRH antagonists for prevention of premature lh surge in ivf/icsi cycles: a randomized controlled trial. *J Family Reprod Health* 2020;*14*:14–20.

Saharkhiz N, Zamaniyan M, Salehpour S, Zadehmodarres S, Hoseini S, Cheraghi L, Seif S, Baheiraei N. A comparative study of dydrogesterone and micronized progesterone for luteal phase support during in vitro fertilization (IVF) cycles. *Gynecol Endocrinol* 2016;*32*:213–217. doi: 10.3109/09513590.2015.1110136.

Siew JYS, Allen JC, Hui CYY, Ku CW, Malhotra R, Østbye T, Tan TC. The randomised controlled trial of micronised progesterone and dydrogesterone (TRoMaD) for threatened miscarriage. *Eur J Obstet Gynecol Reprod Biol* 2018;*228*:319–324. doi: 10.1016/j.ejogrb.2018.07.028.

Siew S, Yan Hui CY, Tan TC, Allen JC, Malhotra R, Ostbye T. Micronized progesterone compared with dydrogesterone for threatened miscarriage: a randomized controlled trial [328]. *Obstetrics & Gynecology* 2015;*125*:104S. doi: 10.1097/01.AOG.0000463637.28791.c1

Taş M, Uludag SZ, Aygen ME, Sahin Y. Comparison of oral dydrogesterone and vaginal micronized progesterone for luteal phase support in intrauterine insemination. *Gynecol Endocrinol* 2020;*36*:77–80. doi: 10.1080/09513590.2019.1655728.

Thongchan S, Phupong V. Oral dydrogesterone as an adjunctive therapy in the management of preterm labor: a randomized, double blinded, placebo-controlled trial. *BMC Pregnancy Childbirth* 2021;*21*:90. doi: 10.1186/s12884-021-03562-6.

Tomic V, Tomic J, Klaic DZ, Kasum M, Kuna K. Oral dydrogesterone versus vaginal progesterone gel in the luteal phase support: randomized controlled trial. *Eur J Obstet Gynecol Reprod Biol* 2015;*186*:49–53. doi: 10.1016/j.ejogrb.2014.11.002.

Yu S, Long H, Chang HY, Liu Y, Gao H, Zhu J, Quan X, Lyu Q, Kuang Y, Ai A. New application of dydrogesterone as a part of a progestin-primed ovarian stimulation protocol for IVF: a randomized controlled trial including 516 first IVF/ICSI cycles. *Hum Reprod* 2018;*33*:229–237. doi: 10.1093/humrep/dex367.

Zargar M, Saadati N, Ejtahed MS. Comparison the effectiveness of oral dydrogesterone, vaginal progesterone suppository and progesterone ampule for luteal phase support on pregnancy rate during ART cycles. *Int J Pharm Res Allied Sci* 2016;*5*:229–236.

Zhang W, Liu Z, Zhang J, Ren B, Liu M, Li J, Zhang W, Guan Y. Comparison of perinatal outcomes of letrozole-induced ovulation and hormone replacement therapy protocols in patients with abnormal ovulation undergoing frozen-thawed embryo transfer: a propensity score matching analysis. *Front Endocrinol (Lausanne)* 2022;*13*:837731. doi: 10.3389/fendo.2022.837731.

**Supplementary Table S1.** Search terms used and number of publications identified for each search string.

| **#ID** | **Search terms** | **Hits  (PubMed®)** |
| --- | --- | --- |
| 1 | "dydrogesteron"[All Fields] OR "dydrogesterone"[MeSH Terms] OR "dydrogesterone"[All Fields] OR "dydrogesterone/adverse effects"[MeSH Terms] OR "20alpha-dihydrodydrogesterone"[All Fields] OR "20alpha-hydroxydydrogesterone"[All Fields] | 772 |
| 2 | "congenital abnormalities"[MeSH Terms] OR ("congenital"[All Fields] AND "abnormalities"[All Fields]) OR "congenital abnormalities"[All Fields] OR("congenital"[All Fields] AND "abnormality"[All Fields] OR "congenital abnormality"[All Fields] OR ("tetralogies"[All Fields] OR "tetralogy"[All Fields]) OR "abnormalities"[MeSH Subheading] OR "abnormalities"[All Fields] OR "abnorma*"[All Fields] OR "malformations"[All Fields] OR "malformation"[All Fields] OR "malformational"[All Fields] OR "malformative"[All Fields] OR "malformed"[All Fields]) OR "malfor*"[All Fields] OR "safety"[MeSH Terms] OR "safety"[All Fields] OR "safeties"[All Fields] | 2,267,519 |
| 3 | ("dydrogesteron"[All Fields] OR "dydrogesterone"[MeSH Terms] OR "dydrogesterone"[All Fields] OR "20alpha-dihydrodydrogesterone"[All Fields] OR "20alpha-hydroxydydrogesterone"[All Fields]) AND (randomizedcontrolledtrial[Filter]) | 159 |
| 4 | postmenopause[MeSH Terms] | 27,085 |
| 5 | (#1 AND #2) OR #3 | 244 |
| 6 | #5 NOT #4 | 181 |

**Supplementary Table S2.** Description of add-on cohort studies.

| **Study and year** | **Study design** | **Country** | **Indication** | **Age of women (years)*** | **No (intervention/control)** | **Intervention (dose and duration)** | **Control (dose and duration)** | **No live birth (Intervention/control), ITT** | **Congenital anomalies** | |
| --- | --- | --- | --- | --- | --- | --- | --- | --- | --- | --- |
|  |  |  |  |  |  |  |  |  | **Coding/ classification** | **When determined** |
| Jiang *et al.,* 2022 | Cohort Study | China | Long term safety in terms of congenital anomalies of LPS | *LPS:* 31.19 (±4.12)  *Short agonist protocol:* 31.48 (±3.91) | 588 (cycles / 1147 cycles) | *LPS:* 225 IU hMG + 2.5 mg Letrozole daily  *Ovulation trigger:* Triptorelin 0.1 mg | *Short protocol cycles:* 0.1mg Triptorelin + 150 IU hMG daily  *Ovulation trigger:* hCG 5000 IU | 752/1445 | International Classification of Diseases 1^st^ revision Q codes | By three years of age |
|  |  |  |  |  |  | Ethinylestradiol 75 mg/d from MC3 onwards; Oestradiol 8 mg/d + DYD 40 mg/d | |  |  |  |
| Huang *et al.,* 2019 | Retro cohort study | China | Neonatal outcomes and congenital malformations in two differed COH protocols | *DYD + hMG protocol:* 31.0 (±4.1)  *Short protocol:*  31.5 (±3.9) | 1151/1724 | DYD 20 mg od + 150–225 IU hMG  *Co-trigger:* 0.1 Triptorelin + 1000 IU hCG | 0.1 Triptorelin + 150–225 IU hMG  *Co-trigger:*  5000 IU hCG | 1429/2127 | International Classification of Diseases 10^th^ revision Q codes | One week after birth |
|  |  |  |  |  |  | *Luteal phase support:* DYD 40 mg/d + MVP 400 mg/d | |  |  |  |
| Zhang *et al.,* 2022 | Retro cohort study | China | Perinatal outcomes of L-OI and HRT protocols in patients with abnormal ovulation undergoing FET | *HRT:* 31.14 (±4.40)  *L-OI:*  30.31 (±4.06) | 6549/1461 | Estradiol valerate 2 mg orally tid | Letrozole 2.5/5mg orally hMG 37.5–75 IU daily (if indicated) + 10,000 IU urinary hCG | 532/555 | Telephone Interview | Not mentioned |
|  |  |  |  |  |  | DYD 20mg daily + Progesterone sustained-release gel vaginal 90 mg until 12^th^ week of pregnancy | |  |  |  |

**Supplementary Table S3.** Summary of overall findings including RCTs and observational studies.

| **“With dydrogesterone” compared to “without dydrogesterone” in the first trimester for the indications of threatened miscarriage or assisted reproduction technologies (ART)** | | | | | | |
| --- | --- | --- | --- | --- | --- | --- |
| **Patient or population:** ART or threatened miscarriage  **Setting:** Clinical  **Intervention:** Dydrogesterone in first trimester  **Comparison:** No dydrogesterone in first trimester | | | | | | |
| Outcomes | Anticipated absolute effects^*^ (95% CI) | | Relative effect (95% CI) | No. of participants (studies) | Certainty of the evidence (GRADE) | Comments |
|  | Risk without dydrogesterone | Risk with Dydrogesterone |  |  |  |  |
| Congenital anomalies (RCT/Cohort) | 34 per 1.000 | 37 per 1000 (25; 57)  3 more  (9 fewer to  23 more) | RR 1.11 (0.73;1.68) | 2680 (8 Studies) | ⨁⨁◯◯ Low^a,b^ | Rate of congenital anomalies is not significantly increased in live births exposed to dydrogesterone in the first trimester |
| *The risk in the intervention group (and its 95% CI) is based on the assumed risk in the comparison group and the relative effect of the intervention (and its 95% CI). CI, confidence interval; RR, risk ratio.  a. Downgraded -1 due to serious limitations in study design (randomization process)  b. Downgrades -1 due to serious imprecision (wide 95% CIs, low number of events) | | | | | | |
| **GRADE Working Group grades of evidence** **High certainty:** we are very confident that the true effect lies close to that of the estimate of the effect. **Moderate certainty:** we are moderately confident in the effect estimate: the true effect is likely to be close to the estimate of the effect, but there is a possibility that it is substantially different. **Low certainty:** our confidence in the effect estimate is limited: the true effect may be substantially different from the estimate of the effect. **Very low certainty:** we have very little confidence in the effect estimate: the true effect is likely to be substantially different from the estimate of effect. | | | | | | |


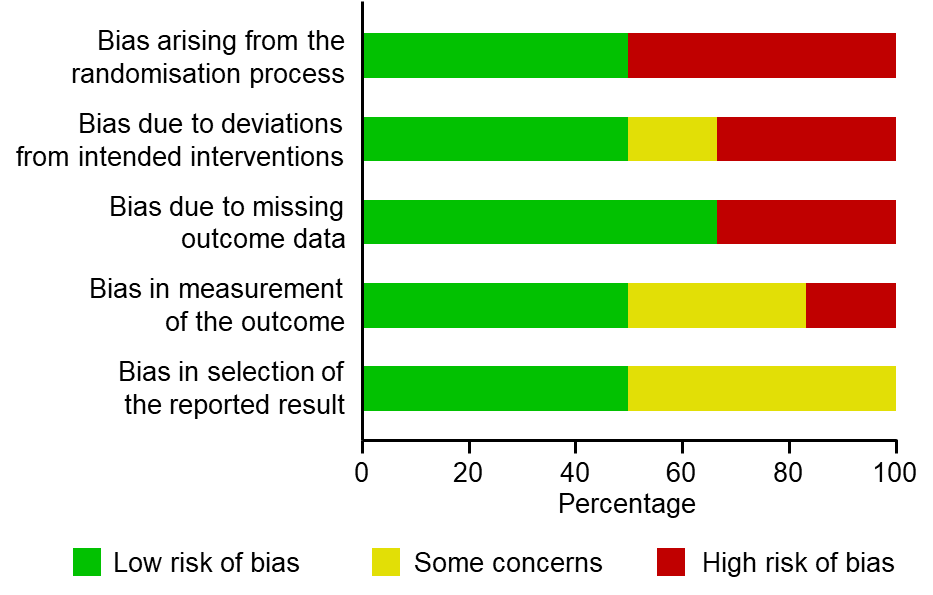


**Supplementary Figure S1.** The risk of bias items presented as percentages across all included randomised controlled trials.


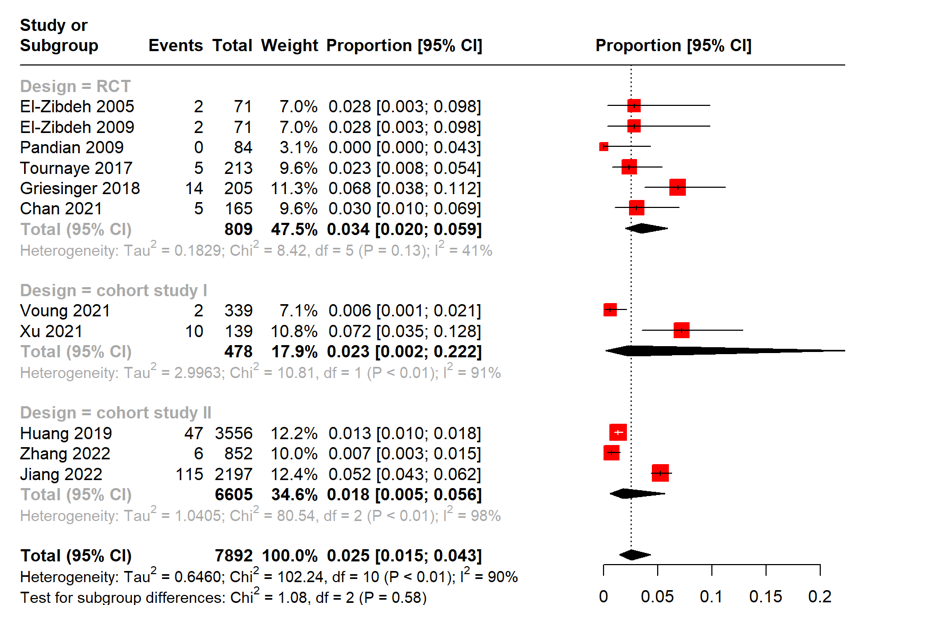


**Supplementary Figure S2.** Meta-analyses of prevalence of congenital anomalies in live births with dydrogesterone exposition in the first trimester, stratified by randomized controlled trials, controlled cohort studies (cohort study I), and non-controlled cohort studies (cohort study II).
